# Parents’ Dental Anxiety in Early Pregnancy and Toothbrushing Stability for Parent and Child Until Age 4—A Longitudinal Study

**DOI:** 10.3390/dj14050271

**Published:** 2026-05-05

**Authors:** Arja Liinavuori, Risto Virtanen, Auli Suominen, Hanna Suokko, Vesa Pohjola, Mimmi Tolvanen, Mika Kajita, Hasse Karlsson, Linnea Karlsson, Satu Lahti

**Affiliations:** 1 Department of Community Dentistry, Institute of Dentistry, University of Turku, 20014 Turku, Finland; risto.virtanen@fimnet.fi (R.V.); vesa.pohjola@utu.fi (V.P.); mika.kaijta@utu.fi (M.K.); satu.lahti@utu.fi (S.L.); 2 Dental Healthcare, Social and Healthcare Services, Western Uusimaa Wellbeing Services County, 02033 Espoo, Finland; 3 Research Unit of Population Health, Faculty of Medicine, University of Oulu, 90570 Oulu, Finland; 4 Emergency Services Academy Finland, 70821 Kuopio, Finland; 5 Department of Clinical Medicine, Turku Brain and Mind Center, FinnBrain Birth Cohort Study, University of Turku, 20014 Turku, Finland; 6 Unit of Child Psychiatry, Department of Psychiatry, Turku University Hospital, University of Turku, 20520 Turku, Finland; 7 Department of Psychiatry, Turku University Hospital, University of Turku, 20520 Turku, Finland; 8 Centre for Population Health Research, Turku University Hospital, University of Turku, 20520 Turku, Finland; 9 Unit of Public Health, Department of Clinical Medicine, Turku University Hospital, University of Turku, 20520 Turku, Finland

**Keywords:** oral health, health behavior, toothbrushing, children, dental anxiety, dental fear, longitudinal survey

## Abstract

Objectives: This longitudinal study examined the association of parents’ dental anxiety during early pregnancy with the stability of brushing their own and their children’s teeth from the age of one to four years. Methods: The study used data from the FinnBrain Birth Cohort Study, which included 816 mothers and 379 fathers who completed questionnaires on dental anxiety at gestational week 14 and on toothbrushing frequency for themselves and their child at the ages of 1, 2, and 4 years. Dental anxiety was assessed using the Modified Dental Anxiety Scale. The stability of toothbrushing was categorized as stable good (twice daily or more at all time points), fluctuating, good at 4 years (fluctuates over time points, but good at age 4 years), fluctuating, poor (less than twice daily) at 4 years (fluctuates over time points, but poor at age 4 years), stable poor (poor at all time points). Unordered multinomial logit models regarding the association of parents’ dental anxiety on brushing their own and their children’s teeth were adjusted for education, and education and parents’ own toothbrushing, respectively. Results: Compared to the mothers who brushed their teeth twice daily throughout the study (“stable good”), those belonging to the “fluctuating, good at 4 years” group and those belonging to the “stable poor/poor at 4 years” group were more likely to have higher dental anxiety (OR = 1.07, 95% CI = 1.01–1.13 and OR = 1.04, 95% CI = 1.00–1.08, respectively). This association was not found among fathers. Parents’ dental anxiety was not associated with the brushing of their children’s teeth. Conclusions: Attending to the mother’s dental anxiety during pregnancy could improve her toothbrushing.

## 1. Introduction

Children’s oral hygiene habits seem to follow those of their parents [1]. Poor oral hygiene habits, on the other hand, often lead to poor oral health [2,3,4,5]. The development of caries can be reduced by establishing a child’s toothbrushing twice a day in early childhood [2,3,4]. Parents who do not brush their own teeth as recommended, that is, less than twice a day, are more likely to brush their children’s teeth less often than recommended [1,6,7,8,9].

Brushing a child’s teeth less than twice a day at the age of 12 months predicted poor toothbrushing when the child was 24 months old [10]. The habit of brushing teeth twice a day acquired in childhood tends to persist into adolescence in most cases [11]. However, changes in brushing a child’s teeth occurred in both directions from age 12 months to 24 months [10]. A mother’s oral hygiene habits and oral health self-care capabilities also affect their children’s toothbrushing frequency [6,8,9]. The better they take care of their own teeth, the more often their children brush their teeth twice a day. A father’s influence on children’s toothbrushing frequency has been studied less, but it also had a significant effect on brushing the children’s teeth [6].

Dental anxiety is a common phenomenon globally [12,13,14,15]. It is estimated that the prevalence of dental fear and anxiety is about 15%, varying from 5 to 42%, and the prevalence of high dental fear and anxiety is about 12%, varying from 4 to 22% [13]. In longitudinal studies, for the majority, dental anxiety remained stable at a low, but for some, dental anxiety increased, decreased, or remained at a stable high over time [14,16,17]. Women experience dental anxiety more often than men [13,14,15], and according to most studies, younger people have dental anxiety more often than older people [12,14,15]. The results on the effect of education on dental anxiety are contradictory [12,14,15,16]. According to some studies, those with lower education report dental anxiety more often than those with higher education [12,15,16].

Of the oral health habits, dental avoidance has been consistently more common in those with dental anxiety [17,18,19,20]. Those with dental anxiety also report greater treatment need [20]. In longitudinal studies, dental avoidance was more common among those who reported increased or stable high dental anxiety than among those who were not at all afraid or whose dental anxiety decreased [17,19]. Of the other oral health habits, toothbrushing has also been associated with dental anxiety [18,21]. Those adults who had high dental anxiety tended to have poorer toothbrushing practices than those with no to moderate dental anxiety. Those who reported high dental anxiety were most likely to brush their teeth less than twice per day. On the other hand, the association between low toothbrushing frequency and having a high dental anxiety is not found in every study [15]. In addition, parents’ dental anxiety has also been shown to affect the dental attendance and dental health of their children [22,23,24]. One reason can be dental shame, which has been associated with dental anxiety, oral health behaviors, and dental attendance [25]. Thus, it is likely that parents’ dental anxiety could affect other oral health habits, like the toothbrushing of the children’s teeth.

However, no studies were found reporting on the association between the stability of brushing habits of the parents and the brushing of their children’s teeth in relation to parents’ dental anxiety. Thus, to fill this knowledge gap, the aims of this study were to analyze whether parents’ dental anxiety during the first trimester of pregnancy was associated with the stability in brushing of their own and their children’s teeth from age one to four years.

## 2. Materials and Methods

This longitudinal study was based on a secondary analysis of the data collected in the FinnBrain Cohort Study, in which the effects of environment and heredity on children’s development and health are studied in an interdisciplinary [26]. The study protocol was approved by the Ethics Committee of the Hospital District of Southwest Finland (14.6.2011 ETMK: 57/180/2011 § 168). The mothers (*n* = 5790) and their partners (later called fathers) attending free ultrasound examinations performed on the mothers at gestational week 12 were recruited for the study between December 2011 and April 2015 at maternal clinics in the South–Western Hospital District area. The fathers who did not participate in the ultrasound appointment were asked by the mothers to participate. Of those invited, 3808 mothers and 2623 fathers, who were expecting 3837 children (twins included), decided to participate and gave their written informed consent.

This study was based on the questionnaire data collected at gestational week 14 (gw 14) for dental anxiety and education; for toothbrushing, the data were collected when the children were 1, 2, and 4 years old. Any parent who had filled in the questionnaire but did not answer the questions on toothbrushing of their own or their child’s teeth at all time points or on dental anxiety at gw 14 was excluded, resulting in 816 mothers and 379 fathers with a corresponding child.

Overall, in the FinnBrain Cohort study [26], those who dropped out of the study were younger, had lower education, and were more often male. Families with older parents and those with higher education were more likely to remain in the study and be included in this study population.

At the 1- and 2-year time points, questions on the frequency of toothbrushing were asked with 7 response alternatives: 3–4 times per day, twice daily, once daily, 2–3 times per week, once a week, twice a month, and seldom or never. At the 4-year time point, toothbrushing frequency was asked with an open-ended question: “How many times you/your child use(s) a toothbrush per day”. All toothbrushing frequencies were dichotomized as good (twice daily or more often) and poor (less than twice daily). Dichotomizations were performed according to Finnish Current Care Guidelines [27]. Variables on the stability of toothbrushing frequency were created based on 1-, 2-, and 4-year questionnaires for mothers’ and fathers’ brushing of their own and their children’s teeth. Stability in the toothbrushing was categorized as follows: Stable good (good at all time points), fluctuating, good at 4 years (fluctuates over time points, but good at age 4 years), fluctuating, poor at 4 years (fluctuates over time points, but poor at age 4 years), and stable poor (poor at all time points). The categorization was based on previous results on changes in toothbrushing between 1 and 2 years [10] and with a specific focus on those who brushed poorly, i.e., not according to recommendations at 4 years. For multivariate modeling categories, Stable poor and Fluctuating, poor at 4 years were combined due to small group size, resulting in three groups for the outcome variable: Stable good, Fluctuating, good at 4 years, and Stable poor or Fluctuating, poor at 4 years.

Dental anxiety was assessed at gw 14. The measurement point for dental anxiety was selected as in Finland, where both parents expecting their first child are eligible, and usually encouraged by mother and child health care, to receive a free estimation of the state of their oral health and need for treatment by an oral health care professional in the first trimester of pregnancy; they are also entitled to subsidized public oral health care [28]. As dental anxiety remained stable for most fathers and mothers [16], this estimation appointment would enable reaching families with dental anxiety and promote their own and their children’s oral health. The assessment was performed with a reliable and validated Finnish version of the Modified Dental Anxiety Scale (MDAS) questionnaire [29,30,31], which included five questions: (1) If you went to your dentist for treatment tomorrow, how would you feel? (2) If you were sitting in the waiting room (waiting for treatment), how would you feel? (3) If you were about to have a tooth drilled, how would you feel? (4) If you were about to have your teeth scaled and polished, how would you feel? (5) If you were about to have a local anesthetic injection in your gum, above an upper back tooth, how would you feel? The response alternatives varied from not anxious (=1) to extremely anxious (=5). A sum score for MDAS was calculated, ranging between 5 and 25, with lower scores indicating lower dental anxiety. For the MDAS sum score, if only one item was missing, it was imputed using the mean value of completed items for that individual. MDAS scores were also categorized into low (sum 5–9), moderate (sum 10–18), and high (sum 19–25) dental anxiety, as high dental anxiety has predicted irregular attendance [17,18,19,20,32].

The confounders were selected based on previous literature [6,10,14,15]. Education was selected as it had the strongest socioeconomic predictive ability for health habits in this population [33]. Education measured at gw 14 was trichotomized as follows: 1 = low (high school/vocational ≤ 12 years), 2 = medium (polytechnics), and 3 = high (university degree or comparable). The categorization was based on both the number of years and the orientation of education according to the Finnish system, which has a compulsory level (9 years), a secondary level with vocational or general/academic (11–12 years) orientation, and a further level with vocational (polytechnics) or academic (university degree or comparable) orientation.

Descriptives were performed as frequencies with percentages for categorical variables and means with standard deviations for continuous variables. Bivariate associations between trichotomized dental anxiety and stability of toothbrushing, and between stability of the own toothbrushing and brushing the child’s teeth, were evaluated separately for mothers and fathers using crosstabulation and chi-squared tests or when test assumptions were not valid with Fisher–Freeman–Halton exact tests. The differences between mothers and fathers were evaluated with a chi-squared test for categorical variables and with a Mann–Whitney U-test for MDAS sum scores.

An unordered multinomial logistic regression model was used in two sets of analyses, separately for mothers and fathers. The first set estimated the association between parents’ dental anxiety at gw 14 and their own toothbrushing patterns adjusted for parents’ education at gw 14. The second set used two-step modeling. Model 1 estimated the association between parents’ dental anxiety at gw 14 and subsequent child toothbrushing patterns adjusted for parents’ education at gw 14. Model 2 additionally included parents’ toothbrushing stability to explore whether estimates changed when accounting for shared family routines (not interpreted as causal pathway effects). Model convergence was confirmed, and cell sizes across outcome categories were inspected. To reduce sparse-data problems, the “stable poor” and “fluctuating poor at 4 years” categories were combined for multivariable models. Participants with valid data were included in the models.

Results of the model were expressed as odds ratios (OR) and their 95% confidence intervals (95% CI). The statistical analyses were conducted using IBM SPSS Statistics for Windows, version 29.0.0.0. and the statistical analysis software SAS version 9.4 (SAS Institute). The statistical significance was considered at *p* < 0.05.

## 3. Results

The mean age of the 816 responding mothers was 31.2 years (SD = 4.2), and for the 379 responding fathers, 33.3 years (SD = 5.2). The majority of the parents reported brushing their own and their children’s teeth at least twice a day at all time points (Table 1). Parents tended to report brushing their own teeth more often than their children’s teeth. Mothers brushed their own teeth more often than fathers did, but there was no difference in brushing their children’s teeth. Most of the parents had low dental anxiety. Dental anxiety was higher among the mothers (Mean = 10.07, SD = 4.38, Median = 9.00, IQR = 5) than among the fathers (Mean = 8.62, SD = 3.76, Median = 8.00, IQR = 4.75) (*p* < 0.001 for Mann–Whitney U-test).

Those mothers who brushed their own teeth at least twice daily at all time points were more likely to report no to low dental anxiety than the mothers whose toothbrushing was fluctuating, good, or poor at the 4-year time point (Table 2). For fathers, such an association was not observed.

There were no differences between the parents’ dental anxiety and the stability of brushing of their children’s teeth (Table 3). Children of those fathers who had high dental anxiety were more often in the stable, good brushing group than children of those mothers who had high dental anxiety.

### 3.1. Parents’ Dental Anxiety and Parents’ Own Toothbrushing

The association between parents’ dental anxiety and their own toothbrushing when adjusted for education was statistically significant for mothers, but not for fathers. Compared to mothers who brushed their teeth twice daily throughout the study (“stable good”), those belonging to the “fluctuating, good at 4 years” group were more likely to have higher dental anxiety (OR for one-point difference in MDAS = 1.07, 95% CI 1.01–1.13). The corresponding ORs (95% CI) for the five-point difference in MDAS score were 1.40 (1.09–1.80) and for the ten-point difference 1.97 (1.18–3.25). Compared to the “stable good” group, those belonging to the “stable poor/poor at 4 years” group were also more likely to have higher dental anxiety (OR for one-point difference in MDAS = 1.04, 95% CI 1.00–1.08). The corresponding ORs (95% CI) for the five-point difference in MDAS score were 1.22 (1.00–1.49) and for the ten-point difference 1.48 (0.99–2.22).

### 3.2. Parents’ Dental Anxiety and Brushing of Their Children’s Teeth

The associations between parents’ dental anxiety and parent-reported brushing of the children’s teeth adjusted for education, and for education and parents’ own toothbrushing (Table 4 and Table 5), were not statistically significant for either parent, while parents’ own toothbrushing had a strong effect on brushing the children’s teeth.

## 4. Discussion

Neither parent’s dental anxiety was associated with brushing their children’s teeth, but mothers’ dental anxiety was associated with their own toothbrushing. Those mothers who had high dental anxiety during pregnancy brushed their teeth more often, less than twice a day throughout the study.

According to our knowledge, the associations between parents’ dental anxiety and the brushing of their children’s teeth have not been studied earlier. The association with mothers’ dental anxiety and their own toothbrushing supports the earlier cross-sectional findings that those who have high dental anxiety more often brush their teeth less than twice a day than those who have no or low dental anxiety [18,21]. On the other hand, among fathers, this association was not found. One reason for this may be the low number of fathers participating in this study. The different roles of fathers have also been shown in relation to dental caries; children of fathers with high dental anxiety had fewer decayed and filled teeth [34]. The strength of the association between the parents’ and children’s dental anxiety has varied for mothers and fathers [35]. On the other hand, only a few studies have reported on the fathers’ dental anxiety and its effect on children’s dental anxiety [34,35,36,37]. Of the confounders, the parents’ own toothbrushing habits were associated with the brushing of the children’s teeth, as found in previous studies on this birth cohort [6,10], but the education was not. When interpreting the results, it must be noted that in this study, mothers with higher education and fathers with lower and higher education were slightly overrepresented among participants compared to the Finnish population [38]. Mothers also seemed to brush their own teeth more often, twice per day, than they reported brushing their children’s teeth. This might indicate that brushing a child’s teeth in early childhood is not always felt as important among mothers, but there could also be many other reasons. Mothers might have been more honest answering the questions, or they can more often be those who brush their children’s teeth than fathers, when fathers are not always aware of their children’s toothbrushing frequency. On the other hand, the agreement between the mothers’ and fathers’ reports on brushing their children’s teeth has been good at ages one and two years [6,10].

The strengths of this study were a longitudinal design and a representative sample of Finnish parents expecting a baby, and the use of validated questionnaires. The study also had several limitations. The number of fathers answering at all time points to the questions of their dental anxiety and their own and their children’s toothbrushing was small, which is one of the limitations of this study. One reason for lower participation rates of the fathers can be that not every father participated in the ultrasonic appointment, and it is not known how many fathers were asked to participate in the study by the mothers [26]. The participants with lower education were more likely to drop out of the study, introducing a potential selection bias due to attrition, which may appear as a better frequency of toothbrushing among the remaining participants. Self-reported questionnaires may also lead to more socially acceptable answers. The different formats for assessing toothbrushing (structured vs. open-ended) at age one and two vs age four might have affected the consistency of the responses. However, the dichotomization according to the recommendations reduced possible inconsistency. The models were adjusted for limited confounders; other confounders could be considered in further studies.

Although parents’ dental anxiety during pregnancy did not affect brushing their children’s teeth in early childhood from age one to four years, it affected mothers’ own toothbrushing. Association between high dental anxiety and brushing one’s own teeth less than twice per day has also been reported in earlier cross-sectional studies for adults and teenagers [18,21,39]. Children usually adopt many of their parents’ health habits and practices [40, 41,42]. Thus, parents’ good toothbrushing habits are important, as parents act as models for the growing child [1,6,7,8,9]. The parents’ model might become even greater when the children start to brush independently without the parents’ help. Though the association between parents brushing their own and their children’s teeth was not observed in this population, future research should investigate if this effect is seen in older children’s own toothbrushing habits. Attending to mothers’ dental anxiety while they are pregnant could improve their own toothbrushing habits and thus, mothers could be a better model for their children’s toothbrushing.

## Figures and Tables

**Table 1:** Distribution (*n*, %) of mothers (*n* = 816) and fathers (*n* = 379) according to their education, stability of brushing their own and their child’s teeth, and dental anxiety in the FinnBrain Birth Cohort.

		Mother		Father		
		*n*	%	*n*	%	*p*-Value
Education (missing 2 + 1)	Low	218	26.7	130	34.4	<0.001
	Medium	230	28.3	120	31.7	
	High	366	45.0	128	33.9	
Toothbrushing (missing 11 + 2)	Stable good	591	73.4	239	63.4	<0.001
	Fluctuating, good at 4 years	71	8.8	38	10.0	
	Fluctuating, poor at 4 years	59	7.2	24	6.4	
	Stable poor	84	10.4	76	20.2	
Brushing child’s teeth	Stable good	419	51.3	193	50.9	0.362
	Fluctuating, good at 4 years	198	24.3	94	24.8	
	Fluctuating, poor at 4 years	73	9.0	24	6.4	
	Stable poor	126	15.4	68	17.9	
Dental anxiety at gw 14	No to low	468	57.4	261	68.9	0.001
	Moderate	296	36.3	104	27.4	
	High	52	6.3	14	3.7	

Abbreviations: good, twice daily or more often; poor, less than twice daily; no to low = MDAS sum 5–9; moderate, MDAS sum 10–18, and high, MDAS sum 19–25; gw 14, gestational week 14. *p*-values for the chi-square test.

**Table 2:** Distribution (*n* (%)) of the stability of parents’ own toothbrushing over four years according to their dental anxiety at early pregnancy in the FinnBrain Birth Cohort Study.

		Stability of Parents’ Own Toothbrushing, *n* (%)				
		Stable Good	Fluctuating, Good at 4 y	Fluctuating, Poor at 4 y	Stable Poor	*p*-Value
Mother’s dental anxiety (*n* = 805)	No to low	349 (59.1)	32 (45.1)	31 (52.5)	50 (59.5)	0.001
	Moderate	217 (36.7)	27 (38.0)	17 (28.8)	30 (35.7)	
	High	25 (4.2)	12 (16.9)	11 (18.7)	4 (4.8)	
Father’s dental anxiety (*n* = 377)	No to low	164 (68.6)	29 (76.3)	19 (79.2)	47 (61.8)	0.571
	Moderate	64 (26.8)	9 (23.7)	5 (20.8)	26 (34.3)	
	High	11 (4.6)	0 (0.0)	0 (0.0)	3 (3.9)	

*p*-values for Fisher–Freeman–Halton exact test; 4 y = 4-year time point.

**Table 3:** Distribution (*n* (%)) of the stability of parent-reported brushing of the child’s teeth over four years according to their dental anxiety at early pregnancy in the FinnBrain Birth Cohort Study.

		Stability of Brushing the Child’s Teeth, *n* (%)				
		Stable Good	Fluctuating, Good at 4 y	Fluctuating, Poor at 4 y	Stable Poor	*p*-Value
Mother’s dental anxiety (*n* = 816)	No to low	247 (58.9)	109 (55.1)	41 (56.2)	71 (56.3)	0.324
	Moderate	152 (36.3)	74 (37.4)	23 (31.5)	47 (37.4)	
	High	20 (4.8)	15 (7.5)	9 (12.3)	8 (6.3)	
Father’s dental anxiety (*n* = 379)	No to low	130 (67.4)	66 (70.2)	19 (79.2)	46 (67.6)	0.564
	Moderate	52 (26.9)	27 (28.7)	5 (20.8)	20 (29.4)	
	High	11 (5.7)	1 (1.1)	0 (0.0)	2 (3.0)	

*p*-values for Fisher–Freeman–Halton exact test; 4 y = 4-year time point.

**Table 4:** Associations of mothers’ dental anxiety in early pregnancy with the stability of brushing their children’s teeth from ages 1 to 4 years: Model 1 (adjusted for education) and Model 2 (adjusted for education and parents’ own toothbrushing) comparison.

		Mother-Reported Brushing of Child’s Teeth (Ref. Stable Good)							
		Fluctuating, Good at 4 Years				Stable Poor/Fluctuating, Poor at 4 Years			
		Model 1 OR (95% CI)	*p*	Model 2 OR (95% CI)	*p*	Model 1 OR (95% CI)	*p*	Model 2 OR (95% CI)	*p*
MDAS (per 1-point)		1.018 (0.979–1.059)	0.369	1.005 (0.964–1.048)	0.817	1.034 (0.995–1.075)	0.085	1.017 (0.973–1.063)	0.462
Education (ref. High)	Low	0.995 (0.659–1.501)	0.980	0.834 (0.539–1.291)	0.415	1.264 (0.829–1.927)	0.276	0.837 (0.510–1.371)	0.479
	Medium	0.797 (0.526–1.207)	0.284	0.693 (0.449–1.070)	0.098	1.349 (0.902–2.018)	0.145	1.058 (0.665–1.683)	0.811
Mother’s toothbrushing stability (ref. Stable good)	Stable poor/Fluctuating, poor and 4 years	—	—	1.777 (0.961–3.288)	0.067	—	—	15.876 (9.746–25.862)	<0.001
	Fluctuating, good at 4 years	—	—	8.119 (4.151–15.879)	<0.001	—	—	6.866 (3.221–14.639)	<0.001

MDAS = Modified Dental Anxiety Scale.

**Table 5:** Associations of fathers’ dental anxiety in early pregnancy with the stability of brushing their children’s teeth from ages 1 to 4 years: Model 1 (adjusted for education) and Model 2 (adjusted for education and parents’ own toothbrushing) comparison.

		Father-Reported Brushing of Child’s Teeth (Ref. Stable Good)							
		Fluctuating, Good at 4 Years				Stable Poor/Fluctuating, Poor at 4 Years			
		Model 1 OR (95% CI)	*p*	Model 2 OR (95% CI)	*p*	Model 1 OR (95% CI)	*p*	Model 2 OR (95% CI)	*p*
MDAS (per 1-point)		0.956 (0.892–1.026)	0.210	0.961 (0.895–1.032)	0.273	0.984 (0.922–1.051)	0.632	0.980 (0.910–1.054)	0.0582
Education (ref. High)	Low	1.051 (0.565–1.956)	0.875	0.883 (0.462–1.687)	0.706	1.245 (0.691–2.241)	0.465	0.843 (0.435–1.632)	0.0612
	Medium	1.584 (0.869–2.889)	0.133	1.582 (0.855–2.929)	0.144	1.024 (0.546–1.919)	0.942	0.800 (0.400–1.600)	0.0528
Father’s toothbrushing stability (ref. Stable good)	Poor	—	—	2.378 (1.230–4.596)	0.010	—	—	9.357 (5.074–17.256)	<0.001
	Fluctuating good	—	—	2.804 (1.296–6.067)	0.009	—	—	1.318 (0.449–3.869)	0.616

MDAS = Modified Dental Anxiety Scale.

## Data Availability

The data presented in this study are available on request from the corresponding author. The data are not publicly available due to privacy and ethical restrictions.
